# The chorioallantoic membrane: A novel approach to extrapolate data from a well‐established method

**DOI:** 10.1002/jat.4271

**Published:** 2021-12-07

**Authors:** Alessandro Maugeri, Giovanni E. Lombardo, Michele Navarra, Santa Cirmi, Antonio Rapisarda

**Affiliations:** ^1^ Department of Chemical, Biological, Pharmaceutical and Environmental Sciences University of Messina Messina Italy; ^2^ Department of Pharmacy‐Drug Sciences University of Bari “Aldo Moro” Bari Italy

**Keywords:** angiogenesis, chick embryo, chorioallantoic membrane (CAM), methods, retinoic acid, toxicological screening, tumor growth, vascular endothelial grow factor (VEGF)

## Abstract

The chorioallantoic membrane (CAM) of the chicken embryo is a highly vascularized extra‐embryonic structure that has been widely used as an in vivo model for the evaluation of angiogenesis. This study was designed to optimize data extrapolation from the most exploited experimental protocol to improve its efficiency and the reliability of the obtainable results. In our study, we followed the most common procedure for CAM assay, employing retinoic acid and vascular endothelial growth factor as standards. CAMs were photographed at *t*_0_, *t*_24_, and *t*_48_; then, the main parameters of the predefined vascular network/area were evaluated. Subsequently, their variations in each CAM were calculated comparing them within the same CAM over the course of the whole treatment (*t*_24_ and *t*_48_), also comparing the treated CAMs respect to the untreated ones. Thus, we provide a novel approach aimed at extrapolating data from CAM assay that allows to (i) have a greater reliability and richness of data; (ii) better estimate the potential pro‐ and anti‐angiogenic activity of new candidate drugs; (iii) save both eggs and time for the experiments.

## Abstract 2

The chorioallantoic membrane (CAM) is a structure exploited for the in vivo evaluation of angiogenesis. This study was designed to optimize data extrapolation from a well‐established experimental protocol to improve its efficiency and reliability of the obtainable results. Thus, we provide a novel approach aimed at extrapolating data from CAM assay that allows to (i) have a greater reliability and richness of data; (ii) better estimate the pro‐ and anti‐angiogenic activity of new drugs; (iii) save both eggs and time for the experiments.

**Abbreviations**
A: average bifurcation angle
ANOVA: analysis of variance
B/mm: number of bifurcations per mm
B/mm^2^
: number of bifurcations per mm^2^
CAM: chorioallantoic membrane
Dm: maximum vessel diameter
RA: retinoic acid
SEM: standard error of the mean
V: vascularization degree
VEGF: vascular endothelial grow factor

## 1 INTRODUCTION

The development of the chicken embryo is accompanied by the sequential appearance of embryonic adnexa: yolk sac, amnios and chorion, and, at last, the allantoid (Sheng, 2010). The latter grows rapidly from the fourth to the tenth day of incubation; during this process, the mesodermal layer of the allantoid merges with the adjacent mesodermal layer of the chorion, thus forming the chorioallantoic membrane (CAM) (Ribatti, 2016). The CAM is formed by a double mesodermal layer and is richly vascularized: the wide network of vessels is connected to the embryonic circulation through allantoid arteries and veins. A rapid capillary proliferation continues until the eleventh day; after that, the mitotic cell division decreases and the vascular system stabilizes on the eighteenth day, just before hatching (Ausprunk et al., 1974). CAM is responsible for the gaseous exchange of the embryo with the outside and absorbs calcium salts, favoring the ossification processes and affecting the embryo and determining the thinning of the shell. Moreover, it stores the waste coming from the embryo avoiding it from accumulating into the albumen.

Among different in vivo models, the CAM is a structure highly appealing to the scientific community for the whole study of angiogenesis, spacing from the mere vascular biology to pharmaco‐toxicological profiling of drugs. Furthermore, this model presents different advantages such as the ease of use and the rapidity of its growth, two elements rarely found in an in vivo experimental model. Nevertheless, so far, the quantification of the angiogenic process of novel candidates from the CAM assay is still heterogenous. Therefore, the aim of this study is to describe a novel approach to extrapolate data from this well‐known procedure in order to obtain more reproducible and accurate data, as well as enhance it in terms of costs and time length.

## 2 MATERIALS AND METHODS

### 2.1 Chorioallantoic membrane assay

**FIGURE 1:**
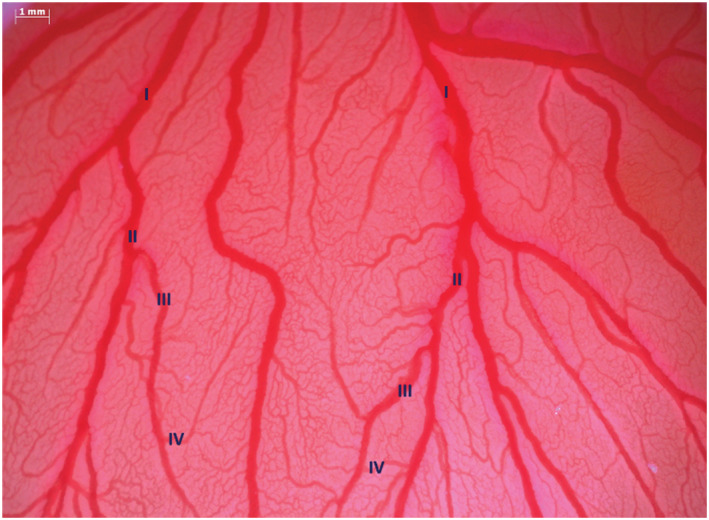
Analyzed area of an example chorioallantoic membrane (CAM) in which the degrees of bifurcation, from the 1st (I) to the 4th (IV), are showed

Fertilized chicken eggs (*Gallus domesticus*) were incubated at 37°C in a controlled humidity, laid horizontally and turned 180° once a day. The fourth day of incubation, a 1 cm^2^ window on the eggshell was performed, careful not to disrupt the remaining structure, after 2 ml of albumen were collected with a syringe. In order to theoretically analyze the same microscopic field in every observation (after 24 and 48 h), a perpendicular line to the central axis of the CAM area was drawn on the eggshell surrounding the window. Afterwards, the development of CAM was assessed and dead or malformed embryos were excluded. Differently from previous methods, eggs were observed and photographed, with Zeiss Stemi 2000‐c stereomicroscope equipped with Axiocam MRc 5 Zeiss camera (Carl Zeiss Microscopy, NY, USA), at the beginning of the experiments (*t*_0_). The degree of vascularization was assessed starting by an allantoid artery, chosen from the perpendicular line set as a reference point, from which the bifurcations from the 1st (I) to the 4th (IV) order were identified (Dimitropoulou et al., 1998) (Figure 1).

### 2.2 Treatments

At *t*_0_ (4^th^ day from incubation), we proceeded with the treatments which were resuspended in the albumen (100 μl) to maintain the physiological condition, and directly applied onto the CAM. Untreated CAMs were provided only albumen (100 μl). Trans‐retinoic acid (RA; Calbiochem, San Diego, CA, USA) was tested at a dose of 2 μg/100 μl per egg (Germano et al., 2015), while vascular endothelial growth factor (VEGF; Sigma‐Aldrich, Milan, Italy) at a dose of 250 ng/100 μl per egg (Wilting et al., 1993). Five eggs were employed for each of the three groups. After each CAM was accordingly treated, eggs were incubated at 37°C for further 24 h (*t*_24_; 5^th^ day from incubation), observed and photographed, re‐incubated for additional 24 h (*t*_48_; 6^th^ day from incubation), then observed and photographed again. The experiment was performed three separate times.

### 2.3 Data analysis

**FIGURE 2:**
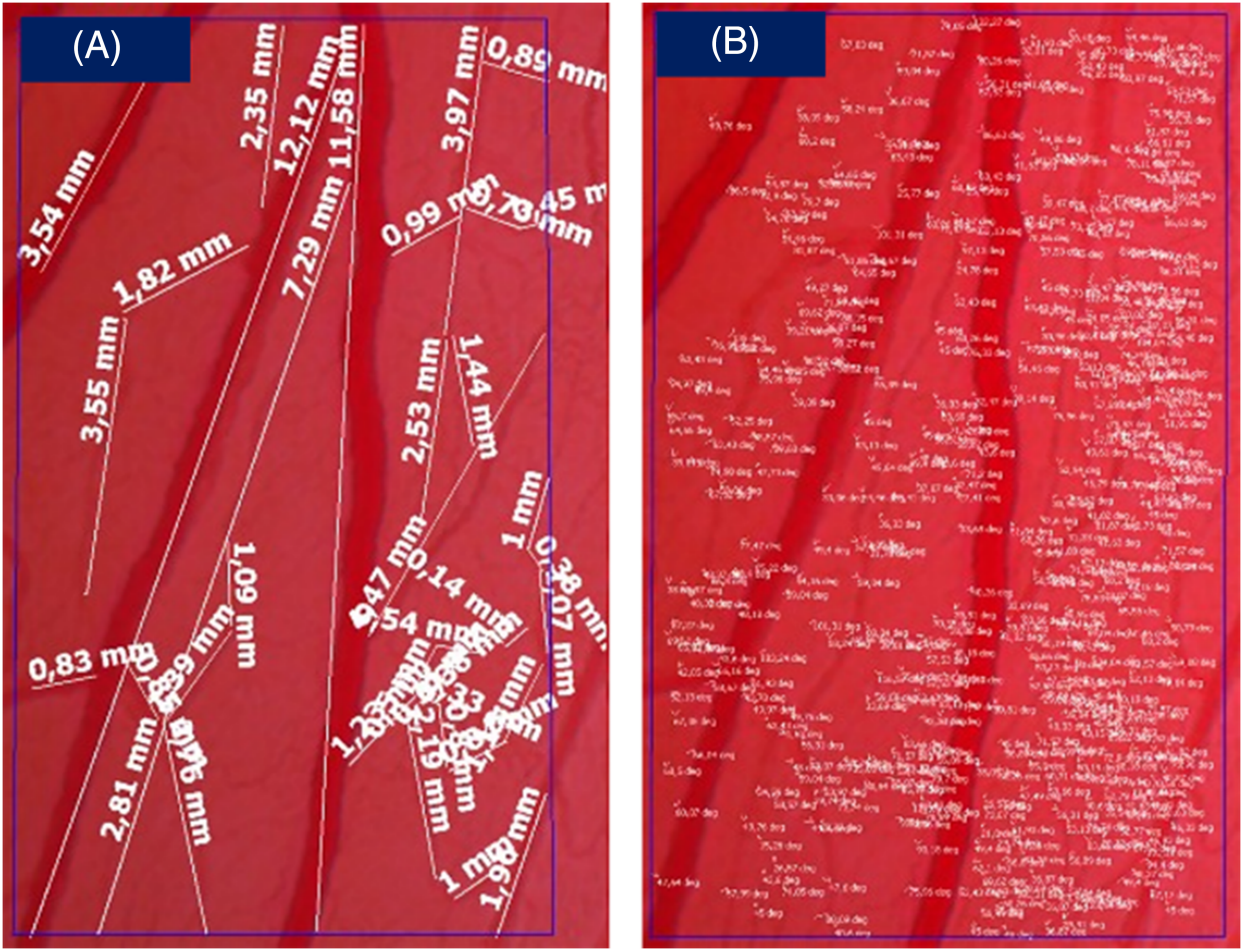
Assessed parameters in the chorioallantoic membrane (CAM). A representative photograph of control CAM is depicted, where area, vessel length (A), number of bifurcations, angle of each bifurcation and average vessel diameter (B) were calculated from tertiary and quaternary vessels

For every microscopic field, the following parameters of tertiary and quaternary vessels and between them were evaluated through the ZEN Blue microphotometric analysis software (Carl Zeiss Microscopy): analyzed area, vessel length, number of bifurcations, angle of each bifurcation and average vessel diameter (Dm; Figure 2). Analyses were performed at the beginning of the experiments (*t*_0_) and after 24 h (*t*_24_) or 48 h (*t*_48_) from the administration of the standards or simply albumen.

From these, derivative parameters were hence calculated:

‐ (V) vascularization degree = vessel length/area;

‐ (B/mm^2^) number of bifurcations per mm^2^ = number of bifurcations/area;

‐ (B/mm) number of bifurcations per mm = number of bifurcations/vessel length.

Percentage changes, for both 24 and 48 h, compared to *t*_0_ within each CAM were calculated according to following formula:

$$\left(\%x\right)\;\text{Percentage change}:\left\{\frac{\left(\text{mean}\;x\;t_{24}-\text{mean}\;x\;t_{0}\right)\;}{\text{mean}\;x\;t_{0}}\times 100\right\}$$

where *x* refers to either V, B/mm^2^, B/mm or Dm.

Percentage changes of treated CAMs respect to untreated ones were calculated according to following formulas:

$$\left(\Delta \%x\right)\;\text{Percentage change}:\left\{\frac{\left(\%x\;\text{treated}-\%x\;\text{untreated}\right)}{\%x\;\text{untreated}}\times 100\right\}$$

where *x* refers to either V, B/mm^2^, B/mm, or Dm.

Furthermore, in order to adequately compare our results to the data present in literature (Song et al., 2003), we expressed them as inhibition percentage, where the antiangiogenic effects of both treatments in the CAM were quantified through counting vessel bifurcations between tertiary and quaternary vessels and expressing this using the following formula:

$$\left\{\text{antiangiogenic activity}=1-\frac{T}{C}\times 100\right\}$$

in which T is the number of vessel bifurcations points in treated CAMs and C is that in untreated ones, in homogenous microscopic fields.

### 2.4 Statistical analyses

The statistical significance of basic parameter raw data was assessed by Student's *t*‐test, considering significant the differences of *p* < 0.05 respect to *t*_0_ and *t*_24_ or *t*_48_ for both untreated and treated eggs (data not shown). The statistical significance of percentage changes of CAMs respect to their *t*_0_ as well as that of treated samples respect to the untreated ones was evaluated through two‐way analysis of variance (ANOVA) with Dunnett's and Bonferroni's multiple comparison test, respectively, considering significant differences for *p* < 0.05, employing GraphPAD Prism 6 (San Diego, CA, USA). Results are expressed as arithmetic mean ± standard error of the mean (SEM).

## 3 RESULTS

**FIGURE 3:**
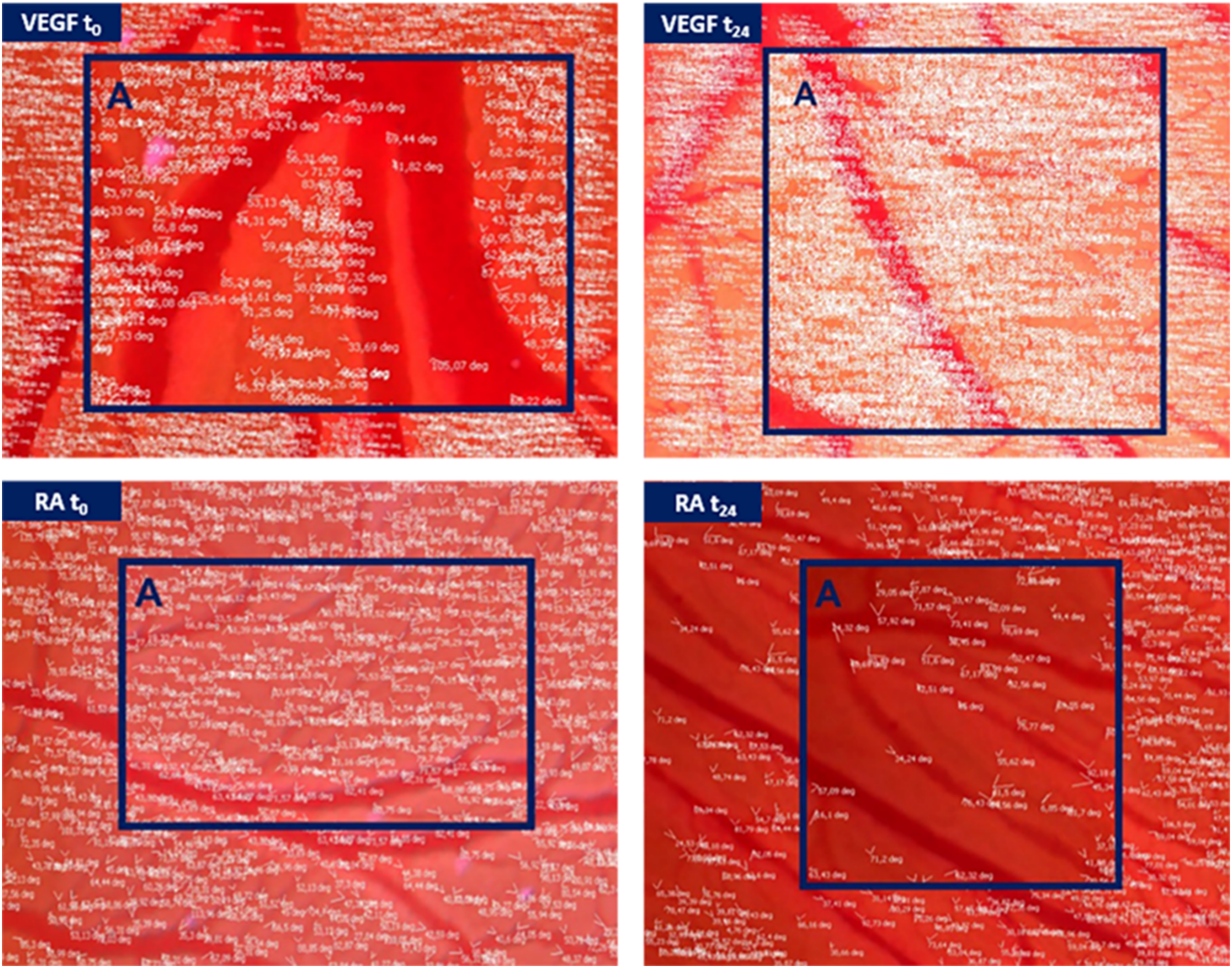
Representative photographs of assessed chorioallantoic membranes (CAMs). Evaluated parameters like area, vessel lengths, number of bifurcations, angle of each bifurcation and average vessel diameter values in vascular endothelial growth factor (VEGF)‐ and retinoic acid (RA)‐treated CAMs of tertiary and quaternary vessels. The blue rectangle defined as (A) represents only a magnification of each CAM

The assessment of this novel approach to extrapolate data from a well‐established procedure started from choosing a proper area to follow the variation of the vessel structure. In Figure 3, an example of an analyzed area of the CAMs is depicted, where afterwards the parameters like area, vessel lengths, number of bifurcations, angle of each bifurcation and average vessel diameter were measured in VEGF‐ and RA‐treated CAMs after 24 h (Figure 3) and 48 h (images not shown) of tertiary and quaternary vessels.

The microscopic analyses were performed at the beginning of the experimental procedures (*t*_0_), and after 24 h (*t*_24_) or 48 h (*t*_48_) from the administration of standards, VEGF and RA, respectively. From the parameters assessed, we firstly evaluated which of these statistically varied from the *t*_0_ employing the Student's *t*‐test, finding that the angle of each bifurcation kept the same trend among untreated and treated CAMs, as well as through various timings. Therefore, we took into account the other parameters of which we calculated the derived ones.

**FIGURE 4:**
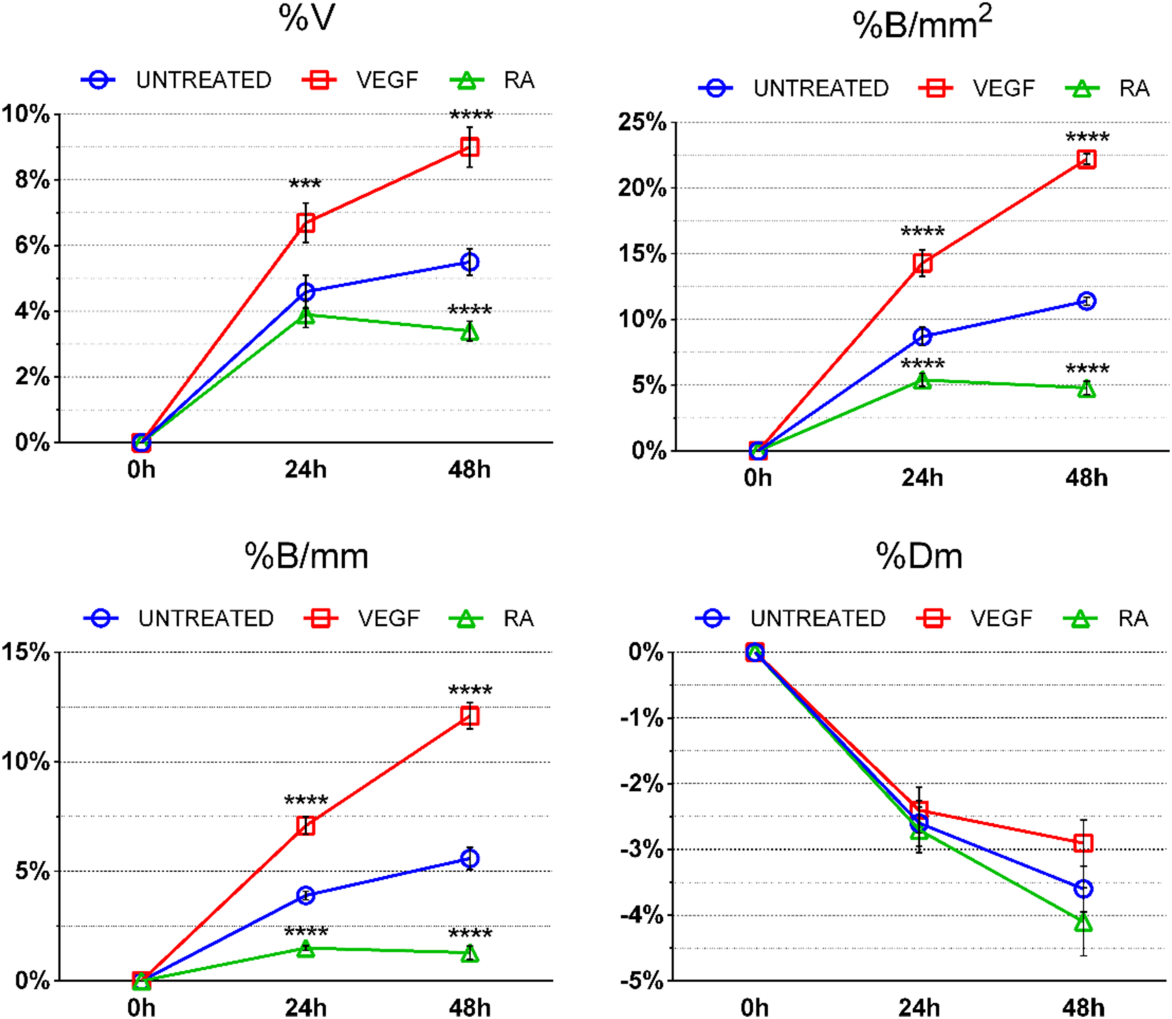
Percentage changes of the parameters evaluated in untreated, vascular endothelial growth factor (VEGF)‐ and retinoic acid (RA)‐treated chorioallantoic membranes (CAMs). The treatment of CAMs with VEGF and RA induced a significant variation of %V, %B/mm^2^ and %B/mm, while %Dm was not significantly affected, respect to untreated CAMs at the same time point. Data are expressed as mean ± standard error of the mean (SEM) of the values obtained from each of the five eggs per group. Experiments were repeated three times. ***p < 0.001; ****p < 0.0001 (VEGF‐ or RA‐treated CAMs vs. untreated ones at the same time point)

The percentage changes from *t*_24_ and *t*_48_ to *t*_0_ of the different parameters assessed showed that untreated CAMs had an increase of %V, B/mm and B/mm^2^ as physiologically expected during angiogenesis, whereas %Dm decreased respect to *t*_0_, due to the increase of number of new capillaries (Figure 4). In both VEGF‐ and RA‐treated CAMs, we observed an increase of angiogenesis, albeit with the obvious variations, respect their *t*_0_. In particular, %V, %B/mm^2^ and %B/mm increased after 24 h and more after 48 h for the VEGF‐treated CAMs respect to the their *t*_0_. Instead, in RA‐treated CAMs, though the increment at 24 h followed the same physiological fashion as seen in untreated CAMs, the whole angiogenic process remained stationary at 48 h, hence clearly hindered by RA treatment (Figure 4). Therefore, the acknowledged pro‐angiogenic effect of VEGF and the anti‐angiogenic one of RA is evident in these data. Finally, %V, %B/mm^2^ and %B/mm in both VEGF‐ and RA‐treated CAMs varied significantly from untreated ones, being great parameters thanks to which it is possible to better appreciate the effects of various treatments through the course of the whole experiment.

**FIGURE 5:**
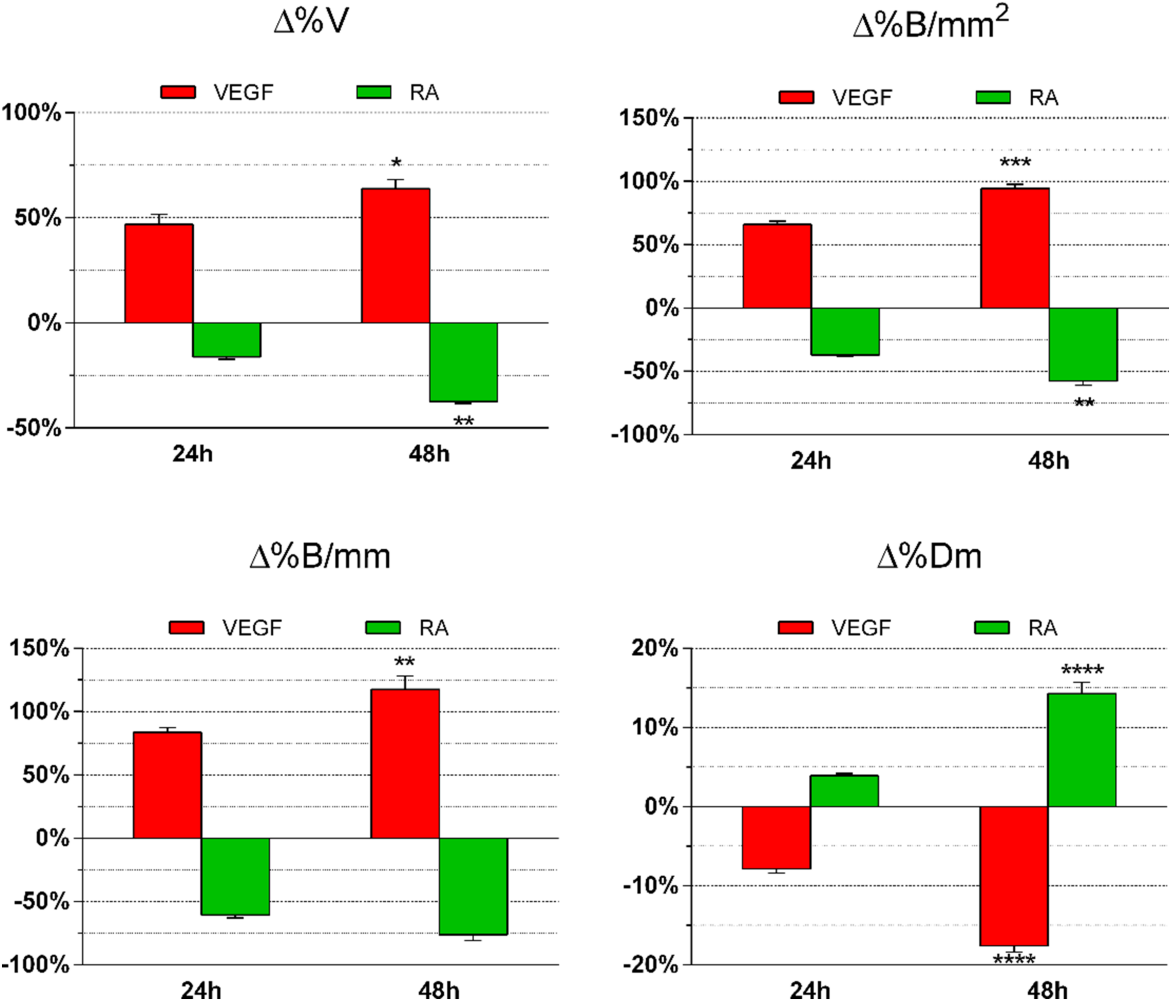
Percentage changes of assessed parameters in treated chorioallantoic membranes (CAMs) respect to untreated ones. The treatment of CAMs with vascular endothelial growth factor (VEGF) increased %V, %B/mm, and %B/mm2, while %Dm decreased. In retinoic acid (RA)‐treated CAMs, the outcome was directly the opposite. Data are expressed as mean ± standard error of the mean (SEM) of the values obtained in each of the five eggs per group. Experiments were performed three times. *p < 0.05; **p < 0.01; ***p < 0.001; ****p < 0.0001 (24 h vs. 48 h)

**FIGURE 6:**
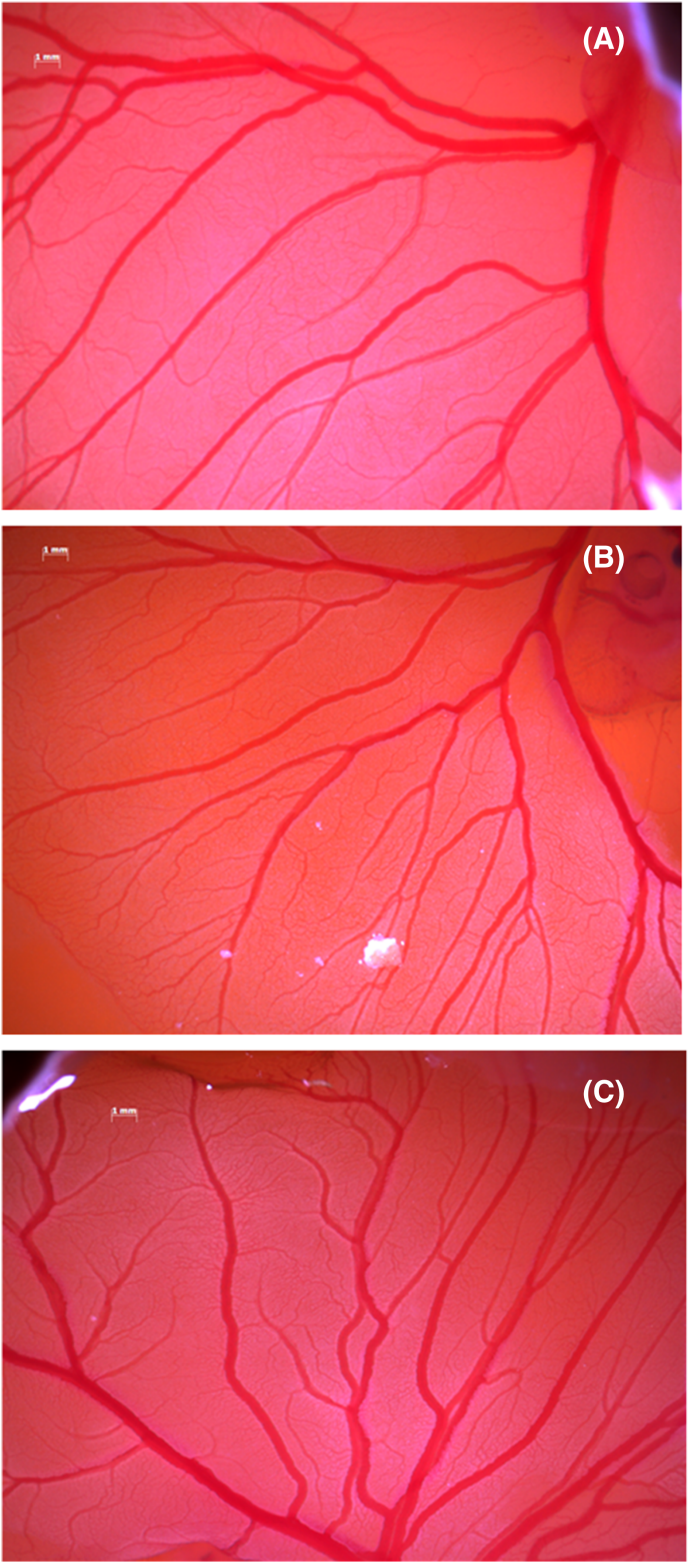
Photographs after 24 h of chorioallantoic membranes (CAMs) untreated (A), treated with retinoic acid (RA) (B) and treated with vascular endothelial growth factor (VEGF) (C)

In order to understand the reason behind the choice of each parameter presented here in this study and that we affirm to be relevant in an in‐depth evaluation of the angiogenesis process, we have further calculated their variation in each treated CAM respect to the untreated one in the same time interval (Figure 5). As seen for the evaluation of the angiogenesis within the same CAMs, the treatment with VEGF increased the degree of vascularization, as well as the number of ramifications per mm and mm^2^, whereas the average vessel diameter decreased. Conversely, the treatment with RA brought to a sharp reduction of the aforementioned values, except for the average vessel diameter that increased. These effects were seen both after 24 h from the treatments (Figure 6), as well as after 48 h with a stronger extent. In particular, in CAMs treated with VEGF, we observed a variation from 24 to 48 h of the degree of vascularization from 47% to 64%, the number of ramifications per mm from 83% to 117% and those per mm^2^ from 66% to 95%, as well as a strong significant decrease of the average vessel diameter from −8% to −18%. In CAMs treated with RA, the degree of vascularization decreased from −16% to −37% and the average vessel diameter significantly augmented from 4% to 14%, whereas the number of ramifications per mm and mm^2^ decreased from −60% to −76% and from −37% to −58%, respectively (Figure 5). Notably, as like the other parameters that varied significantly from 24 to 48 h, %Dm proved to be also another worthy element to be considered when evaluating the variation of the angiogenic process, though significant for longer time points (i.e., from 48 h beyond).

**FIGURE 7:**
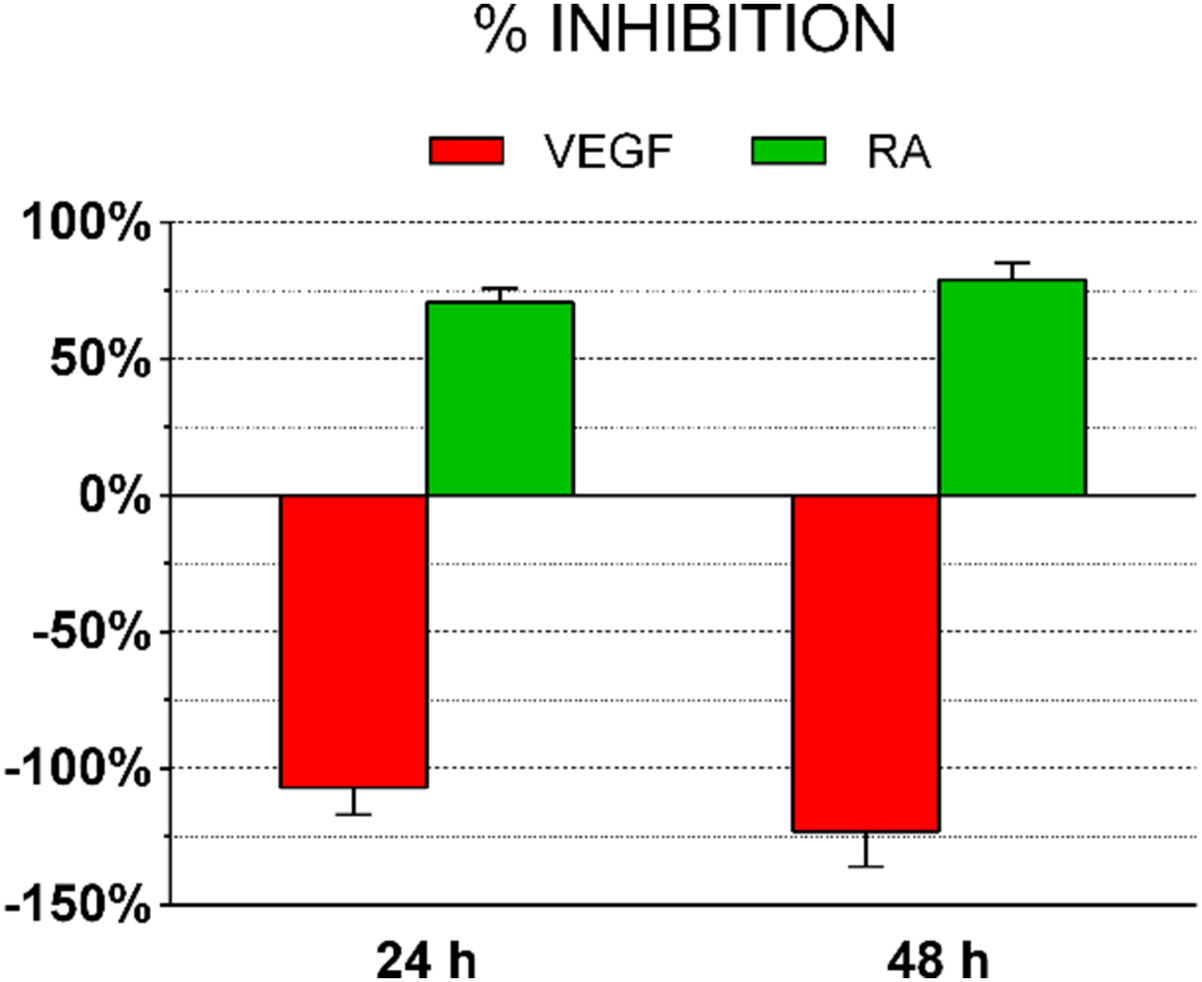
Inhibition percentage of the angiogenesis in treated chorioallantoic membranes (CAMs) respect to untreated ones. The treatment of CAMs with vascular endothelial growth factor (VEGF) increased the number of bifurcations respect to untreated CAMs. In retinoic acid (RA)‐treated ones, the outcome was directly the opposite. Data are expressed as mean ± standard error of the mean (SEM) of the values obtained in each of the five eggs per group. Experiments were performed three times

The most common system for evaluating the anti‐angiogenic effect in CAM is quantifying the number of bifurcations between tertiary and quaternary vessels at the end of the treatments and comparing it to that of the untreated CAMs. Expressing our results as percentage of inhibition of angiogenic activity, we confirmed that they were in line with those widely published in literature (Figure 7). In particular, VEGF stimulated the natural angiogenic process by 107% and 123% at 24 and 48 h, respectively, whereas RA inhibited it by 71% and 79% at 24 and 48 h, respectively. Interestingly, no statistical significance was found between 24 and 48 h employing this formula, thus suggesting that our method may be more accurate for the experimental study of pro‐ or anti‐angiogenic drugs.

## 4 DISCUSSION AND CONCLUSIONS

The CAM assay represents a valid and convenient in vivo test to evaluate the anti‐ or pro‐angiogenic activity of different compounds, from small to more complex molecules (i.e., antibodies) or a mixture of them (i.e., natural phytocomplexes). Additionally, it is even widely used to study the angiogenesis induced by tumor transplants (Komatsu et al., 2019). Moreover, since the rise of the CAM is relatively early in the stage of the development for the chick embryo when it is still not considered as a living being, it does not require ethic committee permission (Ribatti, 2016). Its versatility is due to the fact that the chemical nature of substances to be tested (i.e., solubility or molecular weight) does not represent an obstacle in this procedure since they can be applied onto the CAM through different means, like soaked in synthetic polymers (Langer & Folkman, 1976), directly applied into the site in an albumen‐based solution (Ribatti et al., 1987) or conjugated in a collagen gel between two nylon meshes (Nguyen et al., 1994). Despite these advantages, CAM response in terms of rearrangement of vessels to the abovementioned exogenous materials, choosing the proper duration the testing interval or dealing with the non‐specific inflammatory response when working on a living structure like CAM are elements that researchers need to take into account when planning this type of assay, though being fortunately somehow overcome (Ribatti, 2008). Noteworthy, the yolk sac membrane (YSM) assay is another widely employed method for studying angiogenesis in vivo, which focuses on another anatomical structure of the early developing chick embryo, though being a vessel network as CAM (As et al., 2018; Rosenbruch & Holst, 1990). In particular, the YSM is such another versatile method since substances, both organic and inorganic, can be easily assessed, and hence appreciating their anti‐ or pro‐angiogenic potential (Ma et al., 2016; Wang et al., 2015, 2016).

As like the variety of methods employed to apply substances to study the angiogenetic process in CAM, there are even several semi‐quantitative procedures of evaluating this effect, that always imply the use of a grading scale. Folkman (1974) described that the extent of vascularization should be scored on a graded scale going from 0 to 4. Afterwards, the same author claimed that the measurement scale could range from 0 to 5, from which a ratio of angiogenesis respect to controls can be derived, having 0 when no changes can be seen to 1, which is the maximum rate of vasoproliferative effect (Folkman & Cotran, 1976). Similarly, Knighton and collaborators (Knighton et al., 1977) assessed changes after a graft procedure scoring from 0 to 2 the development of vessels. Some researchers instead counted the number of vessels within a certain area of the CAM after superimposing a ring scale and counting vessels within it (Dusseau et al., 1986) or their length to express it as an index of vessel density (Strick et al., 1991). Nguyen and co‐workers (Nguyen et al., 1994) evaluated the vasoproliferative response counting the squares within a nylon mesh occupied by new vessels and, for anti‐angiogenic agents, compared them to the response to a known angiogenic factor. Ribatti and co‐workers (Ribatti et al., 2001) reported the planimetric method of point counting the new vessels after 12 days from the fertilization through stereomicroscope employing a 144‐point mesh and calculating the intersections with vessels in randomly chosen field. There are also quantitative methods to evaluate this response, as the procedure explained by Elias and Hyde (1983), who numbered the intersections of new vessels to a virtual reticule applied into a photomicroscope ad counted the vessels in 6 random field of the CAM every fixed interval of time, or the employment of newer imaging techniques and agents that allow the identification of vascularization and perfusion along with new vessels formation (Ribatti, 2016). The main characteristics of the most relevant application of the CAM assay are summarized in Table 1.

**TABLE 1:** Characteristics of the most relevant protocols for evaluating angiogenesis through the CAM assay

Reference	Application of the method	Day of the inoculum (from fertilization)	n° of eggs employed	Inoculation technique (drug/tumor)	Quantification of angiogenesis
(Folkman, 1974)	Tumor angiogenesis factor (TAF)	10	Not specified	Pads made of glass fiber filter paper previously impregnated with 5% polyacrylamide gel	Scoring from 0 to 4
(Folkman & Cotran, 1976)					Scoring from 0 to 5
(Knighton et al., 1977)	Tumor‐induced angiogenesis	4–6	632 eggs	Tumor transplant from rats	Scoring from 0 to 2
(Dusseau et al., 1986)	Adenosine‐induced angiogenesis	7 (CAM exposed) 10 (start of experiments)	44 eggs	Ethylene‐vinyl acetate copolymer matrix (Elvax)	Vascular density index (VDI)
(Strick et al., 1991)	Effect of oxygen levels on angiogenesis	7 (1st series: Increasing oxygen concentration) 8–18 (2nd series: Room air experiment)	Not specified (1st series) 61 (2nd series)	Not employed	Evaluation of VDI, vessel length density and vessel endpoint density
(Nguyen et al., 1994)	Quantification of angiogenesis by grow factor or tumor transplantation	8	Not specified	Aluminum sucrose octasulphate and vitrogen mesh	Number of vessels
(Ribatti et al., 2001)	Quantification of angiogenesis by grow factor or tumor transplantation	8	10 per group	Gelatin sponges	Planimetric method of “point counting”

Therefore, in the previous methods on assessing the variation of angiogenesis in the CAM assay, except for the expensive imaging experiments, it was not possible to evaluate the CAM at the *t*_0_ since the number of bifurcations between tertiary and quaternary vessels were only counted in an established area of the untreated CAMs at the end of the experiment in order to compare this value to that of treated and untreated CAMs. Moreover, the expression of results as antiangiogenic activity, hence comparing the number of bifurcations at the end of treatments to those of the untreated CAMs, does not take into account the number of dead embryos in both treated and untreated eggs; therefore, the statistical analyses are quantitatively irregular.

In our experimental procedure, we follow the change of each CAM through the whole course of the experiment, from just prior adding the drug (*t*_0_) to the established time intervals of treatments (*t*_24_ and *t*_48_), thus providing more accurate data, decreasing the intrinsic analytical error when studying such a complex anatomic structure as CAM is. Indeed, a constant increase and development of the vascular network within the CAMs occurs in physiological conditions, effect that is enhanced when we employ a pro‐angiogenic agent (i.e., VEGF) and hindered with an anti‐angiogenic one (i.e., RA). Therefore, it is crucial to follow each individual egg throughout the entire course of the time of the experiment rather than only at defined time intervals (i.e., only at 24 h or 48 h), comparing the degree of vascularization firstly within the same CAM and only after comparing it to that of untreated CAMs. This because, as already pointed out, CAMs can have physiologically denser or sparer vessel network within the same experimental batch, hence giving misleading results in case of not normalizing against the relative *t*_0_, as we propose here. Moreover, although deaths occur through the course of the experimentation, each egg, either treated or not, is compared over itself and hence statistical analyses are based on quantitatively homogenous data. Finally, assisting the micro‐morphometric analysis with a dedicated software, we can obtain more elements like vessel lengths, number of bifurcations and average vessel diameter between tertiary and quaternary vessels, as well as those derived from at each time point in both untreated and treated CAMs, hence making our method more convenient respect the previous ones in terms of time, number of eggs and richness of obtained data. Indeed, given the wide plethora of molecular pathways implied in the angiogenic process, new candidate drugs can interact at different levels and bring different outcomes. Therefore, evaluating more elements of this complex process rather than only the number of bifurcations, our procedure allows to deeply investigate pro‐ and anti‐angiogenic candidates without underestimating their potential, providing a more advantageous procedure to extrapolate data from CAM assay. Notably, our approach can be applied to other in vivo assays (i.e., YSM), which take into account the variation of the vessel network induced by the treatment of novel candidate drugs.

## CONFLICT OF INTEREST

The authors declare that they have no known competing financial interests or personal relationships that could have appeared to influence the work reported in this paper.

## FUNDING INFORMATION

This research did not receive any specific grant from funding agencies in the public, commercial, or not‐for‐profit sectors.
